# Analysis of the role of Ly-1 antibody reactive in different cancer types

**DOI:** 10.1080/21655979.2021.1995100

**Published:** 2021-12-27

**Authors:** Linlin Chen, Congwen Jin, Hao Liu, Rongmei Feng, Zhengdong Li, Jiasheng Zhang

**Affiliations:** a General Surgery, Affiliated Hospital of West Anhui Health Vocational College, Luan, China; b West Anhui Health Vocational College, Luan, China; c Critical Care Medicine, The Lu’an Hospital Affiliated to Anhui Medical University, Luan, China; d Critical Care Medicine, The Lu’an People’s Hospital, Luan, China; e Emergency Surgery, The Lu’an Hospital Affiliated to Anhui Medical University, Luan, China; f Emergency Surgery, The Lu’an People’s Hospital, Luan, China

**Keywords:** LYAR, hepatocellular carcinoma, tcga, prognosis, cancer

## Abstract

LYAR (Ly-1 antibody reactive) is a transcription factor with a specific DNA-binding domain, which plays a key role in the regulation of embryonic stem cell self-renewal and differentiation. However, the role of LYAR in human cancers remains unclear. This study aimed to analyze the prognostic value of LYAR in cancer. In this study, we evaluated the prognostic value of LYAR in various tumors. We research found that, compared with normal tissues, LYAR levels werehigher in a variety of tumors. LYAR expression level was associated with poor overall survival, progression-free interval, and disease-specific survival. LYAR expression was also related to tumor grade, stage, age, and tumor status. Cell counting kit-8, Transwell, and wound healing assay showed that knocking out LYAR significantly inhibited the proliferation, migration, and invasion of hepatocellular carcinoma cells. In addition, this study found that LYARexpression was significantly positively correlated with MKI67IP, BZW2, and CCT2. Gene set enrichment analysis results showed that samples with high LYAR expression levels were rich in spliceosomes, RNA degradation, pyrimidine metabolism, cell cycle, nucleotide excision repair, and base excision repair.

## Introduction

LYAR is a transcription factor with a specific DNA-binding domain (GGTATT/G) [1]. LYAR was first discovered in 1993 and is speculated to be a nucleolar protein that regulates cell growth, embryonic development and reproductive processes [1]. In recent years, studies have found that LYAR plays an important role in the occurrence of human neural tube defect diseases [2], maturation of sperm in male mice [3], biosynthesis of ribosomes [4], translation control [5], and developmental regulation of erythroid cells [6]. It has also been shown that LYAR, PDIA3, NOP14, NCALD, MTSS1 and CYP1B1 are correlated with the prognosis of ovarian cancer [6]. However, there are few studies on the role of LYAR in tumors. Here, the cancer genome atlas (TCGA) was used to study the correlation between LYAR expression and overall survival (OS) rate in 33 cancers and to determine its potential function and prognostic value. We found that LYAR is highly expressed in a variety of cancers and is associated with poor prognosis. Furthermore, we verified the role of LYAR in hepatic cancer cells. LYAR knockdown inhibited the proliferation, invasion, and migration of hepatic cancer cells.

## Materials & Methods

### Cell culture

Human hepatocellular carcinoma cells (HCC cells; HepG2 and HLF) were purchased from the Type Culture Collection of the Chinese Academy of Sciences. Dulbecco’s modified Eagle’s medium (DMEM) supplemented with 10% fetal bovine serum (FBS) and a combination of 1% penicillin and streptomycin was used for the cell cultures.

### Transfection

Lipofectamine® 2000 (Beyotime, Shanghai, China) was used for siRNA transfection according to the manufacturer’s instructions. The transfected RNA was synthesized by Shengong (Wuhan, China). Briefly, diluted solutions of Lipofectamine® and DNA were incubated at room temperature for 20 min. The mixture was added to the cells and incubated for 5 h, and then the cells were cultured in fresh medium supplemented with 10% FBS.

### Western blot analysis

Western blot analysis was performed according to the standard protocols. Forty-eight hours after transfection, cells were lysed with radioimmunoprecipitation assay buffer (Servicebio, Wuhan, China) containing protease inhibitors, and total protein was extracted. Proteins extracted from the hepatic cancer cells were separated by sodium dodecyl sulfate polyacrylamide gel electrophoresis and transferred to a polyvinylidene difluoride membrane. The membrane was incubated overnight with LYAR primary antibody (1:1,000; Elabscience, Wuhan, China) and then with horseradish peroxidase-conjugated secondary antibody diluted with TBST for 2 h at room temperature. The protein bands were visualized using a commercial enhanced chemiluminescence (ECL) kit (Millipore, Billerica, MA, USA) according to the manufacturer’s protocol. ImageJ 1.33 software (National Institute of Health, Bethesda, MD, USA) was used to quantify the protein bands.

### Cell proliferation assay

The Cell Counting Kit-8 (CCK-8, Dojindo, Tabaru, Japan) assay was used to evaluate cell proliferation. After transfection, hepatic cancer cells was seeded in a 96-well plate (1 × 10^3^ cells/well). Then, add 10 μl of CCK-8 solution to each well and incubated for 2 h. The absorbance was measured at 450 nm at 24, 48, 72, and 96 h. These measurements were repeated thrice.

### Transwell assay

The Transwell chamber was used to detect the invasion ability of hepatic cancer cells. Matrigel (50 mg/L at 1:8 dilution) was used to coat the surface of the bottom of the membrane in the upper chamber of the Transwell chamber. HepG2 and HLF cells (2 × 10^3^) were plated in the upper chamber in 200 μL serum-free medium. Complete medium was added to the lower chamber. After 12 h, the cells in the lower chamber were fixed and stained with crystal violet (0.2%). Images were captured with a microscope at 200× magnification and the number of cells.

### Wound healing assay

The transfected hepatic cancer cells were plated in a 6-well plate and, incubated at 37°C with 5% CO_2_until they reached 100% confluence. Thereafter, the monolayer was scratched with a 10 μL pipette tip to create a wound. After removing the cell debris, the continue cells were cultured further under normal conditions. Images of cells were captured 0 h and 24 h, showing the relative distance between the two edges.

### Data mining

The mRNA expression RNASeqV2 data corresponding to of 418 cases in the HCC datase set were downloaded and pre-processed from the TCGA database (https://portal.gdc.cancer.gov//). We also downloaded clinical and prognostic data from the TCGA database. mRNA expression in tumor cell was also downloaded from the cancer cell line encyclopedia (CCLE) database. In addition, mRNA expression in the normal tissues was downloaded from the genotype-tissue expression (GTEx) database.

### Cox regression and survival analyses

Cox regression analysis was used to analyze the correlation between LYAR expression and OS, DSS and PFI of patients with various types of cancers in the TCGA database. After dividing the patients into LYAR high and low expression groups, the Kaplan–Meier method was used to construct survival curves of patients with various types of cancers.

### LinkedOmics

We logged in to the LinkedOmics database (http://www.linkedomics.org/login.php) by selecting the tumor type as ‘HCC’ and the target as ‘LYAR’ from the homepage. The data set was downloaded from the TCGA database to positively and negatively analyze genes related to LYAR expression.

### GSEA

GSEA was performed using the GSEA 4.0.1 software was used for analysis. According to the expression level of ALAS1 mRNA, the sampleswere divided into high and low expression groups.The collection of annotated gene sets of c2.cp.kegg.v7.0.symbols.gmt in MSigDB was chosen as the reference gene sets in the GSEA software. Using 1000 permutations, we obtained the normalized enrichment scores (NES).

### Statistical analysis

In this study, we used the Kruskal–Wallis test to compare LYAR expression levels between different normal tissues and tumor cells. In addition, LYAR expression levels in the tumor and normal tissues were analyzed using the Wilcoxon test. The Kaplan–Meier method was used to assess the association of clinicopathological features with the OS of patients from TCGA. All statistical analyses were performed using R software (version 3.6.2) and GraphPad Prism 8 (GraphPad Software Inc., CA, USA). Image data were collected using ImageJ software, and each experiment was repeated more than thrice.

## Results

### LYAR expression in various tumors

**Figure 1.**
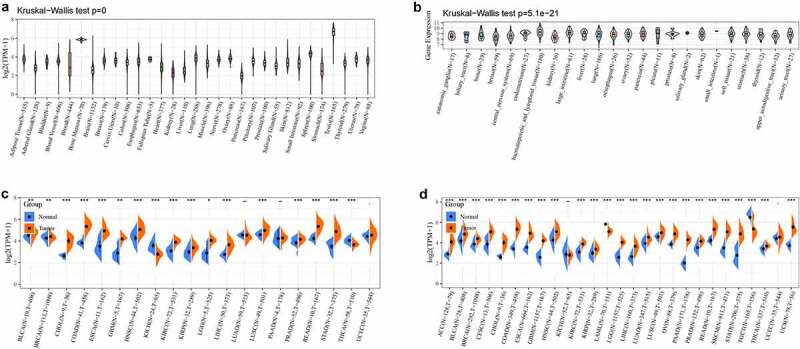
(a) The normal mRNA expression level of LYAR in different tissues from the GTEx database. (b) LYAR mRNA expression levels in various tumor cell lines from the CCLE database. (c) Difference of LYAR mRNA expression between tumor and surrounding samples from the TCGA database. (d) The difference in mRNA expression of LYAR between normal, tumor and tumor samples, combined with data from TCGA and GTEx databases.

We analyzed LYAR the expression in normal tissues using in the GTEx database. LYAR showed the lowest expression in pancreatic tissues and the highest expression in testis tissues (Figure 1a). Analysis of the expression of LYAR in different tumor cell lines in the CCLE database, showed that LYAR was expressed in multiple tumor cell lines, and the expression level was higher in the tumor cell lines than in the normal tissues (Figure 1b). To determine the difference in expression of LYAR between tumor and normal tissues, data corresponding to 20 types of tumors from the TCGA database were analyzed. The results showed that LYAR was highly expressed in most tumors and was only expressed at low levels in KICH (Figure 1c). Since some tumors in TCGA database had insufficient normal matched samples, we combined data from GTEx and TCGA for the analysis. LYAR was only expressed at low levels in the TGCT. These results indicated that LYAR was highly expressed in a variety of tumor tissues (Figure 1d).

### Prognostic value of LYAR in various tumors

**Figure 2.**
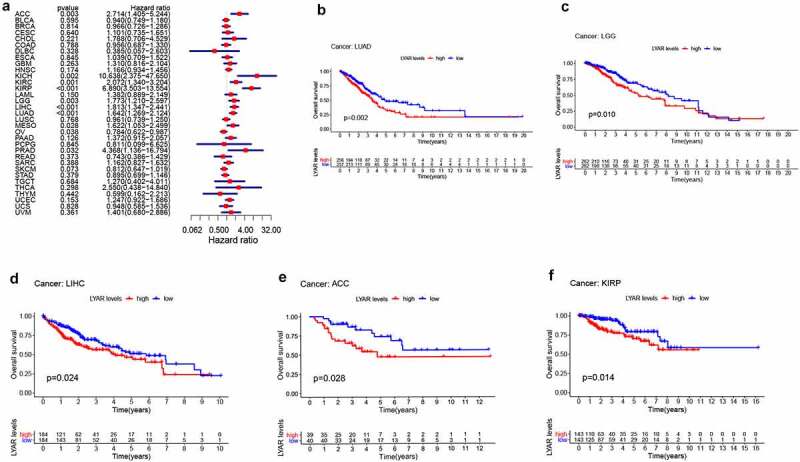
The relationship between LYAR expression and patient OS. (a) Univariate Cox regression analysis showing OS. (b-f) Kaplan-Meier survival curve shows the relationship between the high and low expression of LYAR and the survival rate of patients.

**Figure 3.**
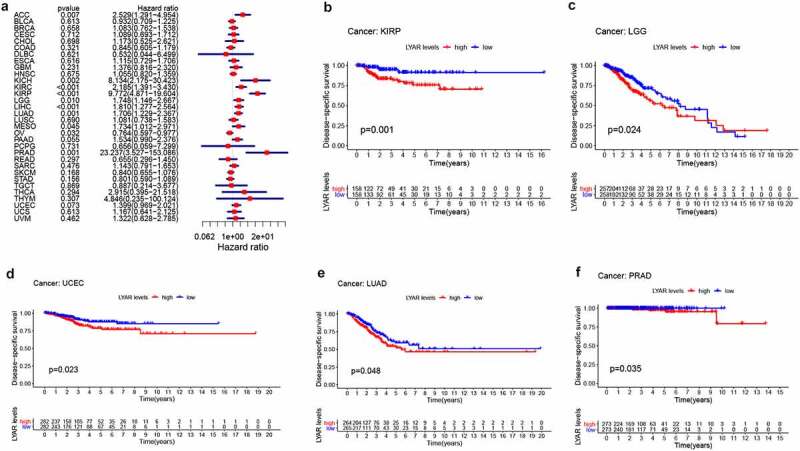
The relationship between LYAR expression and patient DSS. (a) Univariate Cox regression analysis showing DSS. (b-f) Kaplan-Meier analysis of the association between LYAR expression and DSS.

**Figure 4.**
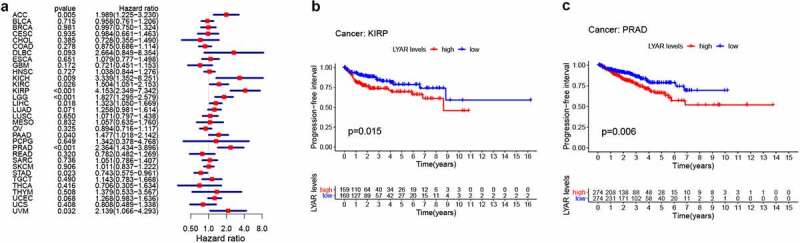
The relationship between LYAR expression and patient PFI. (a) Univariate Cox regression analysis showing PFI. (b-c) Kaplan-Meier analysis of the association between LYAR expression and PFI.

Next, we analyzed the correlation between LYAR expression and OS. Univariate survival analysis of 33 cancer types revealed, that LYAR expression affected the prognosis of KICH, KIRC, KIRP, MESO, PRAD, OV, LIHC, LUAD, LGG and ACC (Figure 2a and Table 1). In addition, the Kaplan–Meier curve showed that among LIHC, KIRP, LUAD, LGG, and ACC, patients with high LYAR expression had a poorer prognosis (Figure 2b–2f). We further analyzed the relationship between LYAR expression and DSS. LYAR expression was found to be related to DSS in LIHC, ACC, KICH, KIRC, KIRP, LGG, MESO, OV, and PRAD (Figure 3a and Table 2). In addition, the Kaplan–Meier curve showed that LYAR was associated with poor prognosis in KIRP, LGG, UCEC, LUAD, and PRAD (Figures 3b–3f). Furthermore LYAR expression was also found to be related to PFI. The forest plot showed that the expression of LYAR is related to ACC, KICH, KIRC, KIRP, LGG, LIHC, PAAD, PRAD, STAD, and UVM (Figure 4a and Table 3). The Kaplan–Meier curve showed that among KIRP and PRAD patients, those with high LYAR expression had a poor prognosis (Figures 3b–3c).

**Table 1.** Univariate Cox regression analysis results show the overall survival of various tumors

cancer	HR	HR.95 L	HR.95 H	p value
ACC	2.714204	1.40473	5.244354	0.002966
BLCA	0.9403	0.749357	1.179897	0.595052
BRCA	0.966309	0.725993	1.286173	0.814277
CESC	1.101286	0.734659	1.650875	0.640429
CHOL	1.787676	0.705665	4.528758	0.220616
COAD	0.955678	0.686532	1.330339	0.788216
DLBC	0.384916	0.056922	2.602877	0.327573
ESCA	1.038828	0.708968	1.52216	0.845058
GBM	1.310457	0.816077	2.104333	0.26319
HNSC	1.166401	0.934092	1.456485	0.174368
KICH	10.63845	2.375181	47.64965	0.001996
KIRC	2.071978	1.339956	3.203905	0.001053
KIRP	6.890246	3.502729	13.55386	2.25E-08
LAML	1.382254	0.889152	2.148817	0.15042
LGG	1.772895	1.210434	2.596719	0.003274
LIHC	1.813166	1.346592	2.441401	8.84E-05
LUAD	1.641775	1.269061	2.123953	0.000161
LUSC	0.961271	0.73937	1.249769	0.768016
MESO	1.622168	1.052815	2.499421	0.028285
OV	0.783603	0.622311	0.9867	0.038095
PAAD	1.371982	0.915089	2.056997	0.125885
PCPG	0.810543	0.099171	6.624701	0.844638
PRAD	4.368089	1.136127	16.79408	0.031896
READ	0.742512	0.385782	1.42911	0.372836
SARC	1.1616	0.826752	1.632067	0.387916
SKCM	0.812285	0.647399	1.019167	0.072501
STAD	0.89502	0.698952	1.146087	0.379326
TGCT	1.269823	0.402005	4.011028	0.683961
THCA	2.549856	0.438129	14.83983	0.297584
THYM	0.59862	0.161917	2.213152	0.441798
UCEC	1.246533	0.92159	1.686047	0.152698
UCS	0.947939	0.584996	1.53606	0.828133
UVM	1.400847	0.679878	2.886357	0.360782

**Table 2.** Univariate Cox regression analysis results show the disease-specific survival rate of various tumors

cancer	HR	HR.95 L	HR.95 H	p value
ACC	2.528918	1.290933	4.954113	0.006845
BLCA	0.931942	0.709151	1.224726	0.613093
BRCA	1.082517	0.762038	1.537776	0.657994
CESC	1.089012	0.692813	1.711786	0.711729
CHOL	1.172707	0.524718	2.620916	0.697814
COAD	0.844508	0.604795	1.179231	0.321135
DLBC	0.532431	0.043622	6.498615	0.621466
ESCA	1.115101	0.728702	1.70639	0.615736
GBM	1.376013	0.816034	2.320262	0.231171
HNSC	1.055475	0.819791	1.358916	0.675389
KICH	8.133926	2.17468	30.42321	0.001844
KIRC	2.184576	1.391277	3.43021	0.000688
KIRP	9.772409	4.871415	19.60416	1.38E-10
LGG	1.748004	1.145701	2.666943	0.00957
LIHC	1.809851	1.27744	2.56416	0.000845
LUAD	1.706013	1.229426	2.367348	0.001395
LUSC	1.080734	0.738027	1.582579	0.689917
MESO	1.734194	1.012211	2.97115	0.045055
OV	0.764045	0.59722	0.977472	0.032253
PAAD	1.533909	0.990124	2.376345	0.055426
PCPG	0.655574	0.058881	7.299027	0.731299
PRAD	23.23745	3.527301	153.0857	0.001074
READ	0.655134	0.296069	1.449663	0.296656
SARC	1.143356	0.790882	1.652918	0.476218
SKCM	0.839833	0.655441	1.076099	0.16756
STAD	0.801116	0.589518	1.088663	0.156457
TGCT	0.887399	0.214137	3.677441	0.869187
THCA	2.914778	0.394833	21.5178	0.294244
THYM	4.846295	0.234575	100.124	0.307027
UCEC	1.399426	0.968996	2.021052	0.073129
UCS	1.167154	0.641046	2.12504	0.61316
UVM	1.322408	0.62799	2.784701	0.462032

**Table 3.** Univariate Cox regression analysis results showed the progression-free interval of various tumors

cancer	HR	HR.95 L	HR.95 H	p value
ACC	1.989474	1.22531	3.230207	0.005408
BLCA	0.957955	0.760617	1.206492	0.715131
BRCA	0.996573	0.750004	1.324204	0.981115
CESC	0.983596	0.661457	1.462622	0.934883
CHOL	0.72755	0.355149	1.490443	0.384687
COAD	0.87451	0.686301	1.114333	0.278165
DLBC	2.663722	0.849307	8.354357	0.092977
ESCA	1.078696	0.776842	1.497842	0.651061
GBM	0.721211	0.450977	1.153373	0.17247
HNSC	1.037564	0.843519	1.276249	0.727034
KICH	3.339486	1.351686	8.250559	0.008975
KIRC	1.504371	1.051321	2.152657	0.025502
KIRP	4.152525	2.348672	7.341791	9.75E-07
LGG	1.827187	1.294613	2.578851	0.000606
LIHC	1.323417	1.049659	1.668573	0.017796
LUAD	1.258149	0.98094	1.613695	0.070541
LUSC	1.070623	0.797102	1.438001	0.650285
MESO	1.056905	0.63457	1.760323	0.831617
OV	0.894397	0.716099	1.11709	0.325188
PAAD	1.476907	1.018231	2.142199	0.039861
PCPG	1.341958	0.377693	4.768031	0.649318
PRAD	2.363692	1.434078	3.895911	0.000741
READ	0.781832	0.481568	1.269313	0.319526
SARC	1.051425	0.785818	1.406807	0.735705
SKCM	1.011491	0.836936	1.222452	0.905899
STAD	0.742868	0.574521	0.960545	0.023392
TGCT	1.14259	0.782808	1.667728	0.489655
THCA	0.705619	0.304766	1.633707	0.41563
THYM	1.378979	0.533127	3.566843	0.507501
UCEC	1.268061	0.982944	1.635882	0.067613
UCS	0.808353	0.488518	1.337588	0.407678
UVM	2.139347	1.065996	4.293457	0.032372

### Correlation between LYAR expression and clinical phenotypes of various cancers

**Figure 5.**
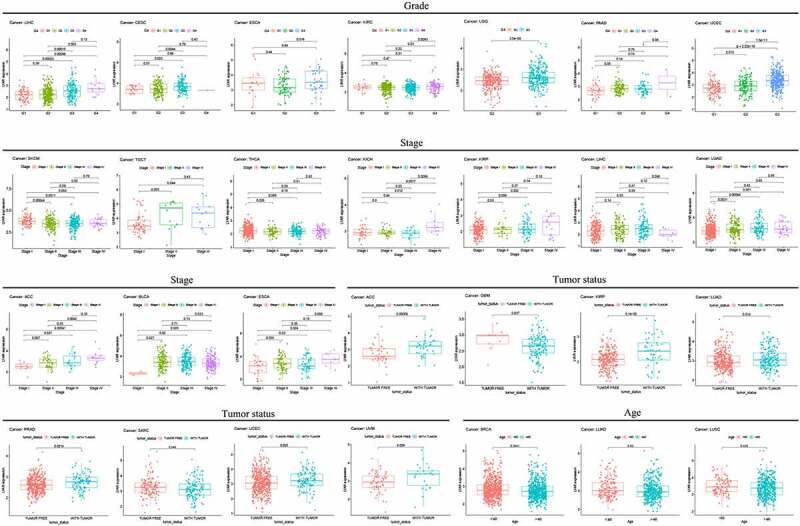
Association with LYAR expression and clinicopathologic characteristics.

LYAR expression was found to be related to tumor grade (Figure 5). In LIHC, CESC, ESCA, KIRC, LGG, PAAD, and UCEC, LYAR expression was higher in the high-grade tumors. In addition, LYAR expression was also related to the stage in SKCM, TGCT, THCA, KICH, KIRP, LIHC, LUAD, ACC, BLCA, and ESCA. LYAR was also related to tumor status. Among KIRP, LUAD, PRAD, UCEC, and UWM, LYAR expression was higher in tumors. However, the expression levels of LYAR in GBM and SARC was low. LYAR expression was also found to be associated with age. Among BRCA, LUAD, and LUSC, lower in patients aged >60 years.

### LYAR promotes migration and invasion of HCC cells

**Figure 6.**
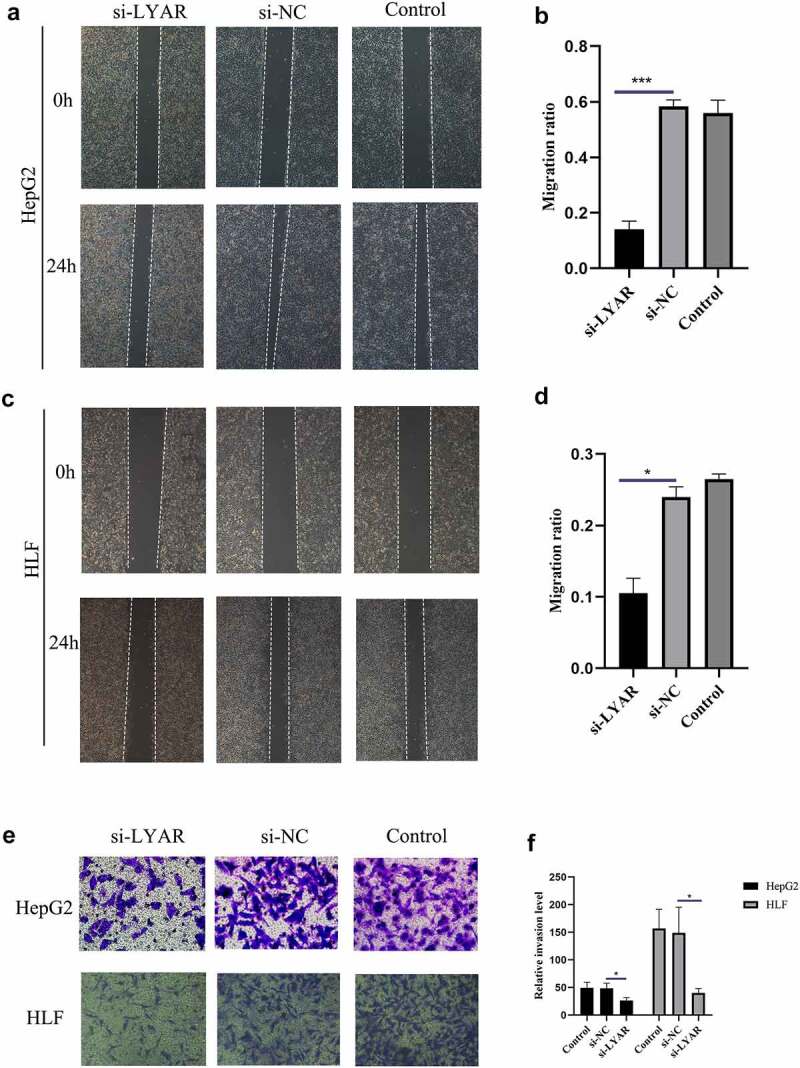
Knockout LYAR can inhibit the growth of HCC cells. (a)Knockout of LYAR reduced the migration ability of HepG2 cells (magnification 40 times). (b)The histogram shows the quantification of HepG2 cell migration. (c)Knockout of LYAR reduced the migration ability of HLF cells (magnification 40 times). (d)The histogram shows the quantification of HLF cell migration. (e) Transwell migration image of HCC cells after LYAR knockout. (f)Transwell cell number of HCC cells after LYAR knockout.

The above analysis revealed that LYAR plays an important role in HCC. Therefore, we analyzed the role of LYAR in HCC cells. In order to study the role of LYAR in HCC cells, we used small interfering RNA (siRNA) to knock out LYAR. We used three siRNA oligonucleotides (siLYAR-1, siLYAR-2, and siLYAR-3) targeting three different regions of LYAR mRNA and found that the protein level of siLYAR-3 was significantly reduced 72 h after transfection (Supplementary Figure). Transwell and wound healing assay were used to study the effect of LYAR on the migration and invasion of HCC cells. Compared with the si-NC group, LYAR knock down significantly inhibited the migration of HepG2 cells (Figures 6a–6b) and HLF cells (Figures 6c–6d). In addition, following after knock out of LYAR, the number of invasive cells was also reduced (p < 0.05) in the knockout group compared to that the si-NC group. (Figures 6e–6f). These data indicate that knocking out LYAR inhibits migration and invasion of HCC cells.

### LYAR promotes proliferation of HCC cells

**Figure 7.**
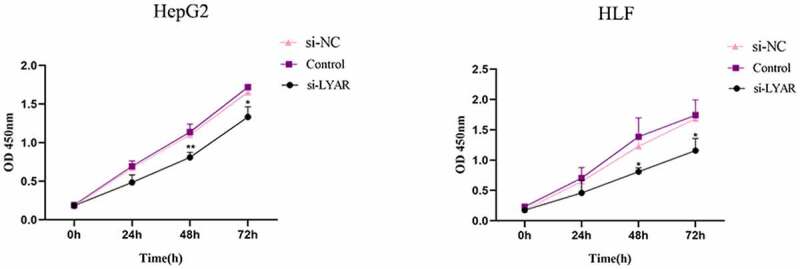
Knockout of LYAR can inhibit HCC cell proliferation.

The CCK-8 assay was used to measure the proliferation of HCC cells. Compared to the si-NC group, the OD value of the si-LYAR group decreased significantly after 48 h and 72 h following transfection (p < 0.05) (Figure 7). This data suggests that LYAR knockdown inhibits proliferation of HCC cells.

### Genes associated with the regulatory network of LYAR in HCC

**Figure 8.**
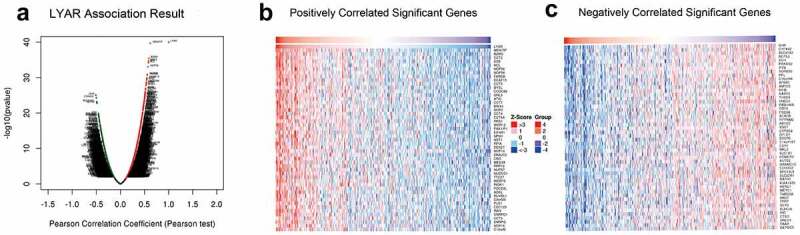
Genes differentially expressed in correlation with LYAR in HCC (LinkedOmics). (a) A Pearson test was used to analyze correlations between LYAR and genes differentially expressed in HCC. (b–c) Heat maps showing genes positively and negatively correlated with LYAR in HCC (TOP 50). Red indicates positively correlated genes and green indicates negatively correlated genes.

**Figure 9.**
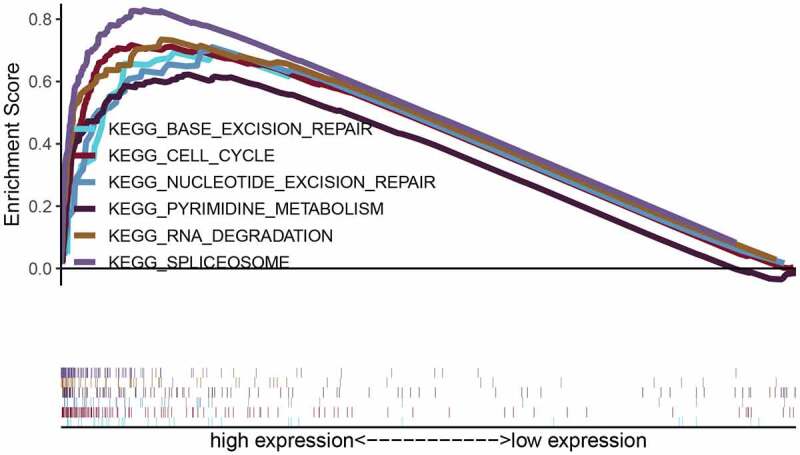
Enrichment plots from gene set enrichment analysis (GSEA). GSEA results showing regulation of spliceosome, RNA degradation, pyrimidine metabolism, cell cycle, nucleotide excision repair, and base excision repair differentially enriched in LYAR-related HCC. NES, normalized ES; FDR, false discovery rate.

Correlation analysis for LYAR was conducted using the LinkedOmics database. With p < 0.001 as the limit, we retrieved 11,255 genes that were positively related to LYAR and 8,667 genes that were negatively related to LYAR (Figure 8a). The top 50 significant genes that positively and negatively correlated with LYAR are shown in the heat map (Figures 8b–7c). Among them, LYAR expression was significantly positively correlated with MKI67IP (r = 0.62, p = 1.90E-40), BZW2 (r = 0.59, p = 3.32E-36), and CCT2 (r = 0.58, p = 2.11E −35), and negatively correlated with GHR (r = −0.51, p = 8.45E-26), and CYP4V2 (r = −0.50, p = 4.71E-25). GSEA was used to analyze the effect of LYAR gene expression level on various biological signaling pathways, and to explore the associated mechanisms (FDR < 0.05, NOM p < 0.05).The GSEA results revealed that samples with high expression of which LYAR were enriched with spliceosome, RNA degradation, pyrimidine metabolism, cell cycle, nucleotide excision repair, and base excision repair (Figure 9 and Table 4). This indicates that LYAR may affect the occurrence and development of HCC by regulating these biological processes or pathways.

**Table 4.** Gene sets enriched in phenotype high

MSigDB collection	Gene set name	NES	NOM p-val	FDR q-val
C2.cp.reactome/biocarta/keg.v7.0.symbols.gmt	SPLICEOSOME	2.197	0.000	0.000
	RNA DEGRADATION	2.133	0.000	0.000
	PYRIMIDINE METABOLISM	2.051	0.000	0.004
	CELL CYCLE	2.007	0.000	0.004
	NUCLEOTIDE EXCISION REPAIR	1.980	0.000	0.004
	BASE EXCISION REPAIR	1.895	0.000	0.013

Note: NES, normalized ES; NOM *p-value*, normalized *p-value*; FDR, false discovery rate.

## Discussion

LYAR is a human ortholog of the mouse nucleolar protein LYAR, a 45 kDa protein containing 379 amino acid residues, including zinc finger motifs and three nuclear localization signals [1]. LYAR is a new type of nucleolar protein with a zinc finger DNA-binding motif, which plays an important role in cell growth and proliferation [1].

Previously, LYAR was found to be upregulated in colorectal cancer tissues, and its expression level is associated with advanced and metastatic colorectal cancer tissues [7]. In neuroblastoma, knocking out LYAR inhibits the proliferation of neuroblastoma cells, and patients with high LYAR expression have a poor OS rate [8]. In our study, we found that LYAR is highly expressed in a variety of cancers and is significantly related to prognosis, grade, and staging. These findings suggest that LYAR plays an important role in tumor development.

Previous studies have shown that LYAR promotes the migration and invasion of colorectal cancer cells by activating the expression of galectin-1 [7]. The expression of galectin-1 is also related to the development of HCC [9]. However, few studies have explored the correlation between LYAR and HCC. Therefore, we analyzed TCGA data to explore the role of LYAR. Our results showed that LYAR was upregulated in HCC tissues and its expression was related to the clinical stage, grade, and vascular tumor cell type. Survival analysis showed that high LYAR expression was related to OS outcomes. In addition, LYAR and five other genes (PDIA3, NOP14, NCALD, MTSS1, and CYP1B1) showed potential as prognostic biomarkers for radical ovarian cancer. Our study also found that LYAR significantly promotes the proliferation, migration, and invasion of HCC cells. These results indicate that LYAR plays an important role in HCC.

The nucleolus is considered to be the region where ribosomal RNA is synthesized, processed, and assembled with ribosomal proteins. The organization and size of the nucleoli are directly related to the production of ribosomes. Therefore, the size of the nucleolus is a diagnostic marker for highly proliferating cancer cells [10]. Studies have shown that nucleolar proteins are involved in RNA metabolism, cell cycle progression, cell proliferation, and apoptosis [11,12]. LYAR is a nucleolar protein that plays an important role in cell growth and proliferation. Through GSEA analysis, we found that samples with high expression of LYAR were significantly enriched in the spliceosome, RNA degradation, pyrimidine metabolism, cell cycle, nucleotide excision repair, and base excision repair among others, and these signaling pathways are related to tumor proliferation [13–15]. Therefore, we speculate that high expression of LYAR may promote the development of HCC by regulating these pathways.

The proliferative ability of embryonic stem cells is closely related to their tumorigenicity [16]. LYAR is highly expressed in embryonic stem cells and plays a key role in the maintenance of embryonic stem cells. When the expression level of LYAR is reduced, the proliferation ability of embryonic stem cells reduces significantly, cell apoptosis increases, and the ability to form teratomas in nude mice is lost [17]. Nucleophosmin is highly expressed in stem cells and tumors, and Mki67ip promotes embryonic stem cell self-renewal by regulating nucleophosmin [18]. As a transcription factor, LYAR can transcriptionally regulate a variety of genes and promote tumor development. Our study found that the expression of LYAR and MKI67IP were significantly positively correlated. We speculate that LYAR may also promote proliferation of embryonic stem cells and promote tumor development through direct transcriptional regulation of MKI67IP or recruitment of MKI67IP. In addition, we found that LYAR expression was significantly correlated with BZW2 and CCT2. BZW2 and CCT2 have been shown to promote the proliferation of various tumors and affect the prognosis of cancer. BZW2 expression has been shown to be of prognostic significance in patients with lung adenocarcinoma [19–22]. Whether LYAR affects HCC growth by regulating these genes requires further in-depth studies.

This study used bioinformatics tools to analyze the expression of LYAR in a variety of tumors and to explore its underlying mechanism in HCC. We provide clues and clinical basis for further research on the role of LYAR in tumors. However, our results were based on a database mining analysis platform and cytology experiments. Therefore, we propose that further animal model experiments should be conducted to delineate the specific mechanism of action of LYAR in tumors.

## Conclusion

All in all, LYAR is highly expressed in most tumors and is related to poor prognosis. In addition, knocking out LYAR can significantly inhibit the proliferation, migration and invasion of hepatic cancer cells. LYAR may participate in the development of HCC by regulating spliceosomes, RNA degradation, pyrimidine metabolism, cell cycle, nucleotide excision repair, and base excision repair.

## Supplementary Material

Supplemental Material

## Data Availability

The following information was supplied regarding data availability: TCGA raw data is available at UCSC xena browser(https://xenabrowser.net/datapages/). Tumor cell line’s data were downloaded from the CCLE database (https://portals.broadinstitute.org/ccle/).

## Research Highlights

- The expression of LYAR in many cancers is higher than that in non-tumor tissues and is associated with poor OS, DSS and PFI.
- LYAR is related to the clinical stage, grade, age, and tumor status of a variety of cancers.
- LYAR can promote the proliferation, migration and invasion of HCC cells.
- LYAR expression is related to spliceosomes, RNA degradation, pyrimidine metabolism, cell cycle, nucleotide excision repair, and base excision repair pathways.

## Abbreviations

LYAR: Ly-1 antibody reactive;
OS: Overall Survival;
DSS: Disease Specific Survival;
DFI: Disease-free Interval;
PFI: Progression Free Interval;
TCGA: The Cancer Genome Atlas;
CCLE: Encyclopedia of Cancer Cell Lines;
GTEx: Genotype-Tissue Expression;
ACC: Adrenocortical carcinoma;
BLCA: Bladder urothelial carcinoma;
BRCA: Breast invasive carcinoma;
CESC: Cervical squamous cell carcinoma and endocervical adenocarcinoma;
CHOL: Cholangiocarcinoma;
COAD: Colon adenocarcinoma;
DLBC: Lymphoid neoplasm diffuse large B-cell lymphoma;
ESCA: Esophageal carcinoma;
GBM: Glioblastoma multiforme;
HNSC: Head and Neck squamous cell carcinoma;
KICH: Kidney chromophobe;
KIRC: Kidney renal clear cell carcinoma;
KIRP: Kidney renal papillary cell carcinoma;
LAML: Acute Myeloid Leukemia;
LGG: Brain lower grade glioma;
LIHC: Liver hepatocellular carcinoma;
LUAD: Lung adenocarcinoma;
LUSC: Lung squamous cell carcinoma;
MESO: Mesothelioma;
OV: Ovarian serous cystadenocarcinoma;
PAAD: Pancreatic adenocarcinoma;
PCPG: Pheochromocytoma and paraganglioma;
PRAD: Prostate adenocarcinoma;
READ: Rectum adenocarcinoma;
SARC: Sarcoma;
SKCM: Skin cutaneous melanoma;
STAD: Stomach adenocarcinoma;
TGCT: Testicular germ cell tumors;
THCA: Thyroid carcinoma;
THYM: Thymoma;
UCEC: Uterine corpus endometrial carcinoma;
UCS: Uterine carcinosarcoma;
UVM: Uveal melanoma;
CCK-8: Cell Counting Kit-8.
