# Supplementary figures and images for: Analysis of the role of Ly-1 antibody reactive in different cancer types
Source: Bioengineered. 2021 Dec 27;12(2):9452–62. doi: 10.1080/21655979.2021.1995100 (PMC8809990; doi:10.1080/21655979.2021.1995100)

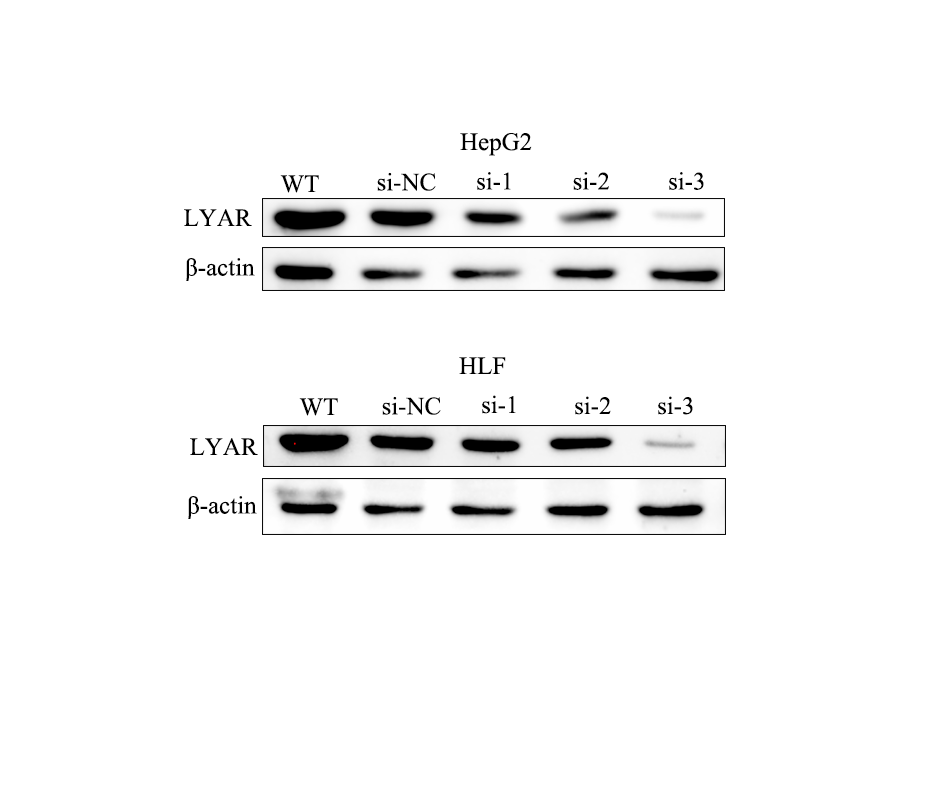

Supplement: Supplemental Material [file KBIE_A_1995100_SM1313.tif]
